# Supplementary material for: Investigating psychobiological causes and mechanisms in functional seizures and functional motor symptoms: Study protocol
Source: PLoS One. 2024 Jun 21;19(6):e0305015. doi: 10.1371/journal.pone.0305015 (PMC11192335; doi:10.1371/journal.pone.0305015)
Supplement: S1 Table — (PDF) [file pone.0305015.s001.pdf]

Laboratory session and remote monitoring phase	
**Dependent variable (rated 1-7 Likert)**	**Item wording: Right now….**
sFunctional motor symptoms	I am experiencing my primary functional motor symptom.
Functional seizures	I am experiencing my primary seizure warning symptom.
Dissociation – depersonalisation	• I feel disconnected from my own body.<br>• l feel separated from what is happening to me.
Dissociation – derealisation	• Things seem unreal to me, as if I am in a dream.<br>• It seems like I am looking at the world through a fog.
Dissociation – amnesia	• I cannot remember things that have recently happened.<br>• I have lost track of what is going on.
Affect – positive	I feel…<br>• Excited<br>• Strong<br>• Proud
Affect – negative	I feel…<br>• Upset<br>• Irritable<br>• Nervous
Arousal	I feel bodily arousal.
Pain	I am in bodily pain.
Fatigue	I feel tired.
**MRI – affective images task**	
**Dependent variable (rated 1-7 Likert)**	**Item wording**
Functional motor symptoms / Functional seizures	FND symptom
Dissociation – depersonalisation	Disconnected
Dissociation – derealisation	Unreal
Dissociation – amnesia	Forgetful
Affect – positive	Happy/Content
Affect – negative	Unhappy/Upset
Arousal	Arousal
Pain	Pain
Fatigue	Tired
