# Supplementary material for: Investigating psychobiological causes and mechanisms in functional seizures and functional motor symptoms: Study protocol
Source: PLoS One. 2024 Jun 21;19(6):e0305015. doi: 10.1371/journal.pone.0305015 (PMC11192335; doi:10.1371/journal.pone.0305015)
Supplement: S2 Table — (PDF) [file pone.0305015.s002.pdf]

Data	Source	Timepoint	Collected by	Purpose	Format
Sociodemographics / medical history	Remote screening interview	Baseline	Member of research team	Eligibility assessment / baseline data	Electronic - MS Office document)
Self-report questionnaire scores	Online questionnaire pack (Qualtrics platform)	Baseline, 3, 6, 12-month follow-up	Participant provides data / Member of research team retrieves data	Assessment of aetiological factors and clinical outcomes	Electronic versions of standardised tools.
Cognitive test scores (standardised)	Computerised and paper-and-pencil tests	Laboratory session	Member of research team	To comprehensively assess cognitive functioning of participants	Electronic records of test performance / paper scoring sheets
Cognitive test scores (experimental)	Computerised tests programmed with experimental software	Laboratory session	Member of research team	To assess mechanistic processes including bodily awareness and attentional biases	Electronic test scores (e.g., E-Prime, Excel)
Psychophysiological measurements	ECG, EDA	Laboratory session	Member of research team	To assess physiological functioning and responses	Electronic output files
Neuroimaging outcomes	Structural and functional brain scans	MRI session	Member of research team / neuroimaging technicians	To assess potential differences in brain structure and function in participants with FND vs controls	Electronic output files
Daily experience ratings	Ecological momentary assessment (LifeData app)	Remote monitoring phase (2-4 weeks)	Participants provide data / member of research team retrieves data	To examine fluctuations in FND symptoms and possible triggers in daily life	Electronic output files (E.g., Excel)
Remote physiological data	Fitbit Charge 5 wearable	Remote monitoring phase (2 weeks-12 months)	Participant provides data / Member of research team retrieves data	To collect long-term physiological data to examine relationships with clinical outcomes	Electronic output files (Excel)
Clinical outcome data	Clinician and patient ratings, in-person or remote	Screening interview, lab / MRI sessions, follow-up phone-calls (3, 6, 12 months)	Member of the research team	To examine changes in clinical status and assess relationships with aetiological factors	Electronic output files (e.g., MS Word, Excel)
